# NUPR1 Promotes Radioresistance in Colorectal Cancer Cells by Inhibiting Ferroptosis

**DOI:** 10.1111/jcmm.70519

**Published:** 2025-04-03

**Authors:** Yimin Fang, Haiyan Chen, Yunhua Liu, Kai Jiang, Yucheng Qian, Jingsun Wei, Dongliang Fu, Hang Yang, Siqi Dai, Tian Jin, Tongtong Bu, Kefeng Ding

**Affiliations:** ^1^ Department of Colorectal Surgery and Oncology (Key Laboratory of Cancer Prevention and Intervention, China National Ministry of Education, Key Laboratory of Molecular Biology in Medical Sciences, Zhejiang Province, China), the Second Affiliated Hospital, Zhejiang University School of Medicine Zhejiang Hangzhou China; ^2^ Zhejiang Provincial Clinical Research Center for CANCER Hangzhou China; ^3^ Cancer Center of Zhejiang University Hangzhou China; ^4^ Department of Radiation Oncology (Key Laboratory of Cancer Prevention and Intervention, China National Ministry of Education, Key Laboratory of Molecular Biology in Medical Sciences, Zhejiang Province, China), the Second Affiliated Hospital, Zhejiang University School of Medicine Hangzhou Zhejiang China

**Keywords:** colorectal cancer, ferroptosis, NUPR1, radiotherapy resistance

## Abstract

Radioresistance is a major clinical challenge and the underlying mechanism has not been thoroughly elucidated. In this study, a radioresistant (RR) cell line is established to explore the transcriptomic signatures of radioresistance in colorectal cancer (CRC). KEGG enriched pathway analysis demonstrated that ferroptosis is inactivated in RR cells. Further detection confirmed that radiotherapy can promote ferroptosis, and ferroptosis inactivation is one of the hallmarks of radioresistance in CRC. What's more, induction of ferroptosis can restore the radiosensitivity of CRC cells. Then, we performed RNA sequencing to compare gene expression between parental and RR cells, and cells pretreated with or without RSL3. Via high‐throughput screening, NUPR1 was identified as a potential candidate for ferroptosis‐mediated radioresistance in CRC. CRC cells can acquire radiation resistance by NUPR1‐mediated ferroptosis suppression in the NUPR1‐overexpressing cell line. More importantly, ZZW‐115, an NUPR1 inhibitor, can sensitise RR cells to radiotherapy. Overall, our findings identify ferroptosis inactivation linked with resistance to radiotherapy. Besides, NUPR1 can promote radiation resistance by inhibiting ferroptosis, and targeting NUPR1 may be a potential strategy to relieve radioresistance associated with ferroptosis in CRC.

**Abbreviations**
CR: complete response
CRC: colorectal cancer
DEGs: differentially expressed genes
DFS: disease‐free survival
FRGs: ferroptosis‐related genes
GO: gene ontology
IR: ionising radiation
KEGG: Kyoto Encyclopedia of Genes and Genomes
OS: overall survival
qRT‐PCR: quantitative real‐time PCR
RR: radioresistant
WB: Western blotting

## 1 Introduction

According to the latest data released by the World Health Organization, the number of new cases of colorectal cancer (CRC) worldwide in 2018 was 1.8 million, accounting for about 10% of the total number of cancer cases, and ranking third. The number of deaths was 862,000, accounting for about 9% of all cancer deaths, and ranking second [1]. Radiotherapy is a mainstay of CRC treatment, which is very effective as an adjuvant therapy to reduce the chance of cancer recurring and spreading. Neoadjuvant chemoradiotherapy followed by surgery and adjuvant chemotherapy has become the standard treatment for Stage II‐III rectal cancer [2, 3, 4, 5, 6]. What is more exciting, radiotherapy can become a curative modality for low rectal cancer patients who achieve complete response (CR) to chemoradiotherapy, thus preserving anorectum function and improving quality of life. Besides, sensitivity to neoadjuvant chemoradiotherapy is closely related to prognosis in CRC. The 5‐year disease‐free survival (DFS) and overall survival (OS) in patients with pathological CR are 83.3% and 87.6%, which are significantly higher than those of patients who did not achieve pathological CR (DFS: 65.6%, OS: 76.4%) [7, 8]. However, less than 25% of patients can achieve a pathological CR from conventional radiotherapy [7, 8]. Radioresistance (RR) is a major clinical challenge and significantly limits the therapeutic efficiency. Elucidating its underlying mechanisms and enhancing sensitivity towards radiotherapy is vital to improve the prognosis of CRC patients.

The ability of ionising radiation (IR) to kill cancer cells relies mainly on its DNA damaging effects, including oxidative base damage, abasic site, single‐strand break and double‐strand break. When these damages cannot be repaired in time, it will lead to cell cycle arrest and cell death [9, 10, 11]. The molecular mechanism of innate or acquired RR is polymodal and associated with a number of biological alterations both within the tumour [12] and in the neighbouring cells that make up its microenvironmentPEVuZE5vdGU [13]. For example, the repopulation of cancer stem cells can escape from radiation‐induced cell death and render RR, which is the main cause of treatment failure [14]. Moreover, the autophagy process can be used by cancer cells to delay apoptotic death and contributes to RR [15]. In addition, hypoxic tumours are more resistant to IR, which can be explained by the oxygen fixation hypothesis [16]. However, the signatures of CRC with RR phenotype are too complex to be clarified and require further investigation.

Recently, it has been demonstrated that IR can induce ferroptosis in lung cancer, ovarian cancer and fibrosarcoma [17, 18, 19]. Ferroptosis is an iron‐dependent regulated cell death caused by excessive lipid peroxidation [20, 21] and the increase in intracellular lipid peroxidation levels is also considered an important marker of ferroptosis [22]. Furthermore, it has been observed that the increase in lipid peroxidation levels serves as one of the mechanisms by which radiotherapy eradicates tumours [19]. Ferroptosis can be induced by endogenous and/or exogenous pathways [23]. The endogenous pathways are mainly activated by blocking intracellular antioxidant enzymes, such as glutathione peroxidase and GPX4. The exogenous pathway is initiated by activating ferroportin or inhibiting transporters on the cell membrane, such as the cystine–glutamate transporter [24]. IR induces ferroptosis in tumour cells by downregulating the expression of SLC7A11, which is a key inhibitor of the ferroptosis pathway, through the ATM pathway [18]. Besides, ferroptosis plays a crucial role in IR‐induced cell death and tumour suppression [17]. Small‐molecule ferroptosis agonists [25, 26, 27] or nano‐ferroptosis agonists [28] can be used alone or in combination with other treatments to kill cancer cells. For example, ferroptosis agonists can be used in combination with cisplatin for head and neck cancer cells [29], and can inhibit the progression of melanoma cells combined with PD‐L1 monoclonal antibodies [30]. In addition, acquired drug resistance of tumours has been reported to be correlated with ferroptosis and can be reversed by inducing ferroptosis [31, 32].

NUPR1, a small, highly basic transcriptional regulator, has been found to be highly expressed in many stress responses [33]. High NUPR1 expression levels have been identified in various cancers including oral squamous cell carcinoma, breast cancer, lung cancer, multiple myeloma, liver cancer and kidney cancer [34]. Xiao et al. found that knockdown of NUPR1 inhibits the proliferation, migration and invasion of breast cancer cells [35]. In the study by He et al., NUPR1 promotes the development of clear cell renal cell carcinoma by activating the PTEN/AKT/mTOR signalling pathway and enhancing the stemness of tumour cells [36].

However, it is currently unclear whether radiotherapy can induce ferroptosis, and whether the inactivation of ferroptosis is involved in the regulation of radioresistance in CRC. At the same time, whether NUPR1 plays a role in this process is unknown. Therefore, this study aimed to explore signatures of CRC with RR phenotype and investigate the regulatory role of ferroptosis in RR of CRC and its underlying mechanisms.

## 2 Materials and Methods

### 2.1 Cell Culture and Establishment of Radioresistant Cells

The human CRC cell line RKO and DLD1 were obtained from the American Type Culture Collection (Rockville, MD, USA). A radioresistant RKO cell line (RR) was constructed by repeated radiotherapy. Cells were initially exposed to 4 Gy of X‐rays for three times. Then, these cells were exposed to 6 Gy of X‐rays for three times. Finally, surviving cells were further exposed to 8 Gy of X‐rays for another three times. The total radiation dose was 54 Gy, which was close to the radiation dose received by patients with rectal cancer in clinical practice. All these cells were cultured in RMPI‐1640 medium (Gibco, California, USA) supplemented with 10% fetal bovine serum (Gibco) in a cell incubator containing 5% CO_2_.

### 2.2 RNA Sequencing and Pathway Enrichment Analysis

Total RNA from RR cells and RKO cells treated with or without RSL3 (a ferroptosis agonist, 1 μM, selleck, S8155, Houston, USA) for 24 h was extracted and subjected to Illumina (GENEWIZ, Suzhou, China) for RNA sequencing. The DEGseq R package was used to analyse and screen for differentially expressed genes (DEGs) with |logFC| ≥ 1.5, FDR < 0.05 in each group. We then applied the “limma” and “clusterProfiler” packages to perform gene ontology (GO) analysis and Kyoto Encyclopedia of Genes and Genomes (KEGG) pathway enrichment analysis.

### 2.3 Quantitative Real‐Time PCR

Total RNAs were isolated from cells with RNA Isolation Kit (Vazyme, Nanjing, China) and were reverse transcribed to cDNA by HiScript II Reverse Transcriptase (Vazyme). Quantitative PCR was conducted using SYBR‐Green PCR kits (YEASEN, Shanghai, China) and a 7500 Fast Real‐Time PCR System (Life Technologies, Shanghai, China). All the primers used were listed in Table S1. β‐Actin was used in quantitative real‐time PCR (qRT‐PCR) for normalisation.

### 2.4 Western Blotting

Western blotting (WB) was performed as previously described [37, 38]. The expression of protein was analysed by WB using primary antibodies against the following target proteins: GPX4 (1:5000, Abcam, ab125066, Cambridgeshire, UK), ACSL4 (1:10000, Abcam, ab155282), SLC7A11 (1:5000, Abcam, ab175186), NUPR1 (1:1000, Abcam, ab6028), GAPDH (Huabio, HA721136, HangZhou, China). Proteins were analysed using ImageJ (National Institutes of Health, Vision 1.53e). GAPDH was used as an internal control. Each experiment was conducted in triplicate.

### 2.5 Cellular Lipid Peroxidation Assay

Cells were seeded in six‐well plates and cultured for 24 h, followed by radiotherapy. After that, the cells were cultured for 24 h, and then the irradiated cells were collected and washed with PBS. 5 μM BODIPY 581/591 C11 dye (Invitrogen, California, USA) was added to each sample. After incubation for 30 min at 37°C, the cells were washed again with PBS and prepared into a cell suspension. A CytoFLEX flow cytometer (BeckmanCoulter, California, USA) was used to analyse the lipid peroxidation levels of thecells.

### 2.6 Cell Apoptosis and Necrosis Assay

Hoechst 33342/PI (propidine iodide) apoptosis and Necrosis Assay Kit was purchased from Beyotime (Shanghai, China). Cells were seeded in six‐well plates and cultured for 24 h followed by radiotherapy. After that, the cells were cultured for 24 h, and then the irradiated cells were collected and washed with PBS. Then, 5 μL of Hoechst 33342 staining solution and PI staining solution were added in. After incubation at 4°C for 20–30 min, the cells were washed again with PBS and analysed by flow cytometry.

### 2.7 Clonogenic Survival Assay

Two hundred to six hundred cells per well were seeded in 12‐well culture plates. Cells were pretreated with drugs (RSL3 100 nM), Lip‐1 (5 μM, selleck, S7699), ZZW‐115 (1 μM, MCE, HY‐111838A, New Jersey, USA), Apoptosis inducer (Beyotime, C0006S) and Necrosis inducer (Beyotime, C1058S) or DMSO for 24 h followed by radiotherapy. Then, the 12‐well plates were placed in a cell incubator with 5% CO_2_ for 9–14 days. The medium containing drugs or DMSO was replaced every 2 or 3 days. After the cells were cultured for 9–14 days, the medium was removed. Then, 0.5 mL of methanol was added to the well to fix the cells. Next, the plates were placed on ice for 30 min. The fixed cells were stained with crystal violet (Beyotime) for 20 min. The surviving fraction was normalised to that of unirradiated control cells.

### 2.8 Transmission Electron Microscope

Cells were collected in 1.5 mL EP tubes and fixed with 2.5% glutaraldehyde solution overnight at 4°C. Then, cells were washed with PBS gently three times; 1% osmium tetroxide‐PBS was added for fixation for 1 h. The fixed samples were dehydrated in a graded ethanol series. Then, cells were critical point dried and sputter‐coated with 10% gold. Cell morphology was observed using a transmission electron microscopy (TECNAI10, Philips, Amsterdam, Holland).

### 2.9 Subcutaneous Tumorigenesis Model

Balb/c athymic nude mice (SLAC Laboratory Animal, Shanghai, China) were maintained and subjected to the experiments in accordance with the protocols approved by the Second Affiliated Hospital of Zhejiang University School of Medicine Animal Care and Use Committee. Specifically, 5 × 10^5^ RKO parental or RR cells in 100ul PBS were injected subcutaneously on the back of the mice. When tumours grew to approximately 100 mm^3^, mice were randomised to experimental groups and received radiotherapy (8 Gy for twice). Tumours were measured every 2–3 days, and the tumour volume was calculated as 1/2 × length × width^2^. Then, tumours were harvested 7 days after radiotherapy for further analysis.

### 2.10 Ionising Irradiation of Cells and Subcutaneous Tumours

Cells or subcutaneous tumours were irradiated with an X‐ray beam from a linear accelerator (Siemens, Munich, Germany), giving the cells or mice a highly uniform and precise dose of radiation. The linear accelerator produces an X‐ray photon beam (6 MV) with a dose rate of 3 Gy/min.

### 2.11 Lentivirus Production and Infection

The lentivirus overexpressing NUPR1 was purchased from Miaoling Bio (Wuhan, China), including pLV3‐CMV‐hNUPR1‐FLAG‐CopGFP‐Puro and its control vector (pLV3‐CMV‐hNUPR1‐FLAG‐CopGFP‐Puro). When the cells have grown to around 60%–70% confluency, the medium was changed to complete medium containing lentiviral particles (MOI = 5–20) and polybrene (2–5 μg/mL). After 24 h, the lentivirus‐containing medium was replaced with fresh complete medium. After culturing for 48 h, 2 μg/mL puromycin was used for screening to obtain the stable transfected cell line. The overexpression of NUPR1 in the stable transfected cell line was verified by WB.

### 2.12 Public Database Mining

Gene expression microarrays and corresponding clinical information of 243 rectal cancer patients who received radiotherapy were downloaded from the Gene Expression Omnibus (GEO) (GSE87211, https://www.ncbi.nlm.nih.gov/geo/query/acc.cgi?acc=GSE87211). After excluding patients with incomplete information, a total of 142 patients were included in the analysis, of which 91 were divided into the Response group (patients with tumour remission after chemoradiotherapy) and 51 were divided into the non‐Response group (patients without remission or tumour progression after chemoradiotherapy). These patients were randomly assigned to the training and testing sets at a ratio of 7:3.

The Lasso‐Logistic regression analysis was used to get the genes that are related to the response to chemoradiotherapy in CRC from 214 ferroptosis‐related genes (FRGs). These FRGs are taken from the data sets of ferroptosis driver genes, suppressor genes and marker genes downloaded from FerrDb (Table S2) [39]. LASSO algorithm uses ‘glmnet’ R package for variable selection and contraction. The independent variable in the regression was the normalised expression matrix of known FRGs [39], and the response variable was the tumour regression of patients receiving chemoradiotherapy. The ‘pROC’ R package was used to conduct ROC curve analyses to evaluate the predictive power of the gene signature. The ‘survminer’ package in R was used to search for the optimal cut‐off expression value based on the risk score.

Gene expression profiles and clinical data from patients in the MSK‐Colon cohort were downloaded from cBioportal (https://www.cbioportal.org/study/summary?id=rectal_msk_2022) [40]. A total of 69 patients were divided into the Response group (patients with decrease in T stage after radiotherapy, *N* = 49) and the non‐Response group (patients with unchanged T stage after radiotherapy, *N* = 20). NUPR1 expression was further investigated and compared in these two groups. Besides, the correlation between NUPR1 and known ferroptosis suppressor genes [39] in CRC was explored in TCGA tumour by GEPIA (http://gepia.cancer‐pku.cn/).

### 2.13 Statistical Analysis

All data are presented as the mean ± SD or mean ± SEM. Data collection and analysis were performed using GraphPad Prism 8.0.2. The data between two groups were analysed by two‐tailed unpaired Student's *t*‐test, and the data of more than two groups were analysed by ANOVA. *p* < 0.05 was considered statistically significant.

## 3 Results

### 3.1 Transcriptomic Signatures of Radioresistant CRC Cells

**FIGURE 1:**
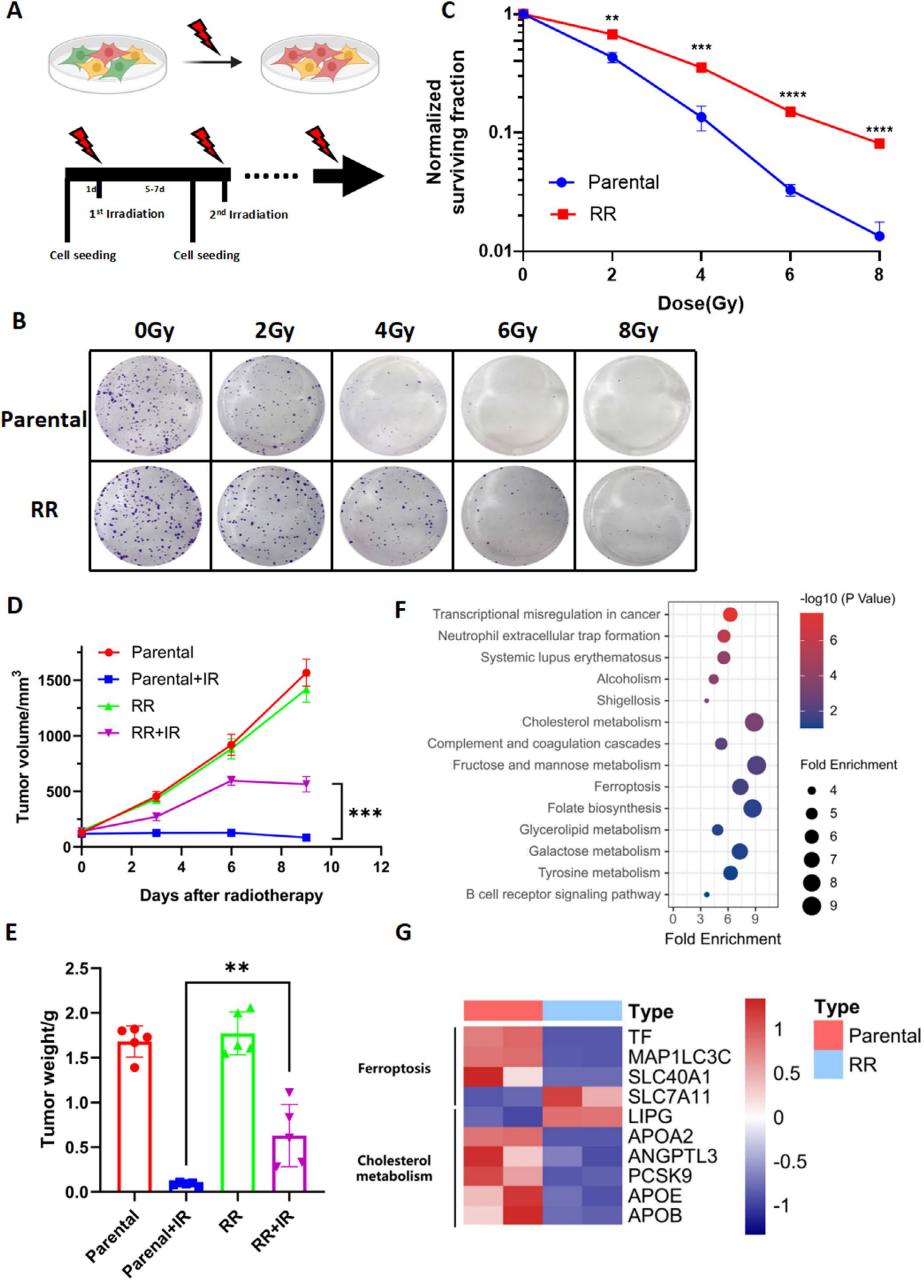
Transcriptomic signatures of radioresistant CRC cells. (A) Flow chart of induction of RKO‐RR cells. (B, C) Clonogenic survival curves for RKO cells and RKO‐IRresist cells receiving radiotherapy (from 0 to 8 Gy). The survival data were normalised to those of unirradiated cells. *n* = 3, **p < 0.01, ***p < 0.001, ****p < 0.0001 by two‐tailed unpaired Student's *t*‐test. (D, E) In subcutaneous tumorigenesis model, RKO and RR tumour growth (D) and tumour weight (E) after radiotherapy (8 Gy twice). *n* = 5, **p < 0.01, ***p < 0.001 by two‐tailed unpaired Student's *t*‐test. (F) Kyoto Encyclopedia of Genes (KEGG) pathway enrichment analysis. (G) Heatmap of RNA sequencing between Parental cells and RR cells.

In order to understand the characteristics of CRC with a radioresistant phenotype, we established a radioresistant CRC cell line (RR) by repeated challenge of parental RKO cells with irradiation (Figure 1A). The colony‐forming ability of RR cells was significantly higher than that of parental cells when exposed to a single dose of radiation up to 8Gy (Figure 1B,C). Besides, BALB/c nude mice were injected subcutaneously with parental and RR cells and locally irradiated by X‐ray (Figure S1A). As expected, the size of parental tumours was dramatically reduced after radiotherapy, whereas that of RR was not (Figure 1D,E and Figure S1B,C). Collectively, these results demonstrated that this radioresistant CRC cell line was successfully developed and validated.

Then, we investigated the transcriptomic signatures of RR cells by RNA sequencing. PCA analysis revealed a difference between parental and RR cells (Figure S1D). We found 129 significantly upregulated DEGs and 86 significantly downregulated DEGs. KEGG pathway enrichment analysis demonstrated that DEGs were significantly enriched in pathways including transcriptional misregulation in cancer, neutrophil extracellular trap formation, cholesterol metabolism, fructose and mannose metabolism and ferroptosis (Figure 1F). Besides, GO analysis of biological processes was categorised into cellular process, single‐organism process, and biological regulation (Figure S1E). Within the cellular components category, most of the DEGs were enriched in cell and cell part. Likewise, GO molecular functions analysis showed that DEGs were important in binding and catalytic activity. Other significant items in biological process, cellular components and molecular functions are listed in sequence in Figure S1E.

### 3.2 Ferroptosis Inactivation Is One of the Hallmarks of Radioresistance in CRC

**FIGURE 2:**
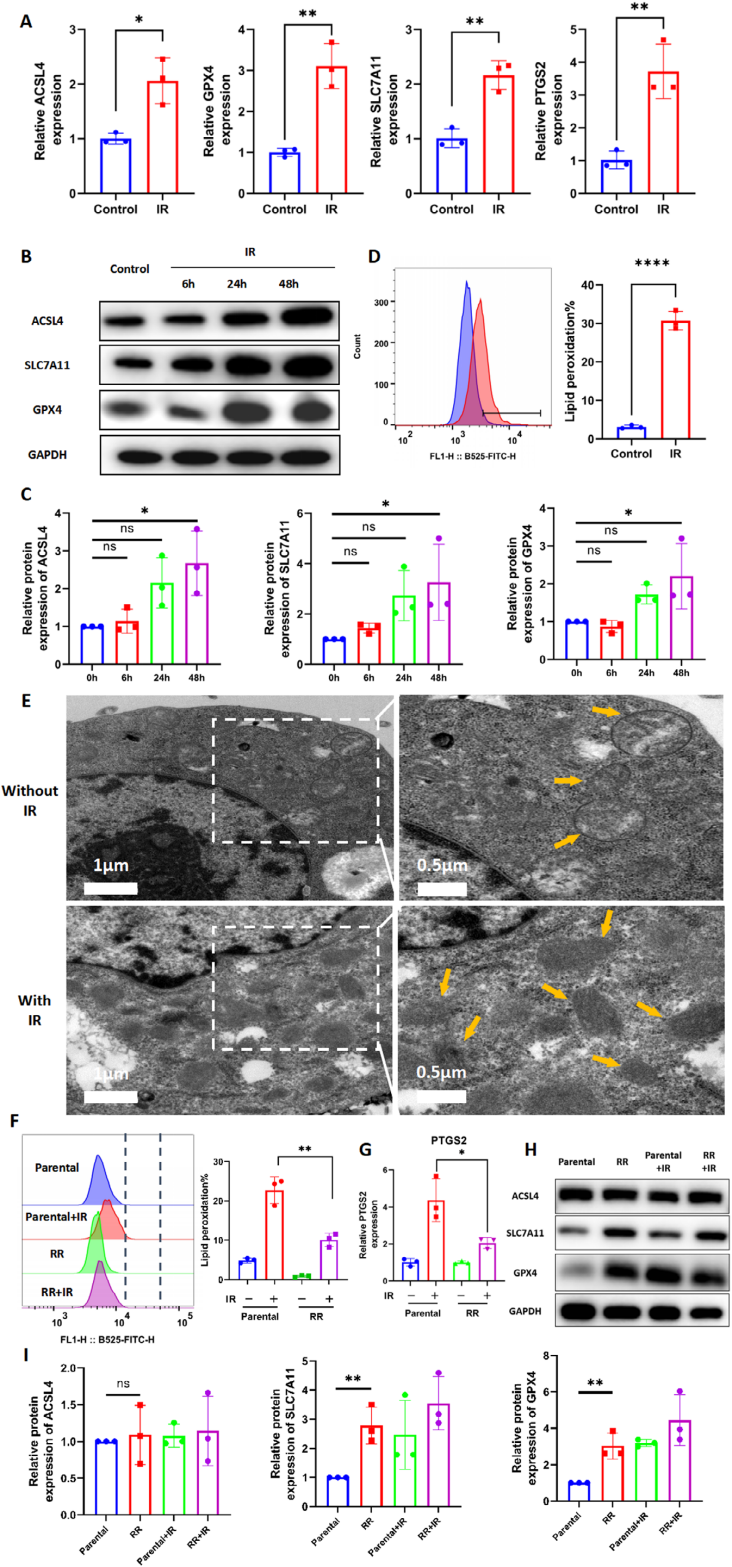
Ferroptosis inactivation is one of the hallmarks of radioresistance in CRC. (A) qRT‐PCR analysis of ACSL4, GPX4, SLC7A11 and PTGS2 in RKO cells at 24 h after receiving radiotherapy (6 Gy). *n* = 3, *p < 0.05, **p < 0.01, ****p < 0.0001 by two‐tailed unpaired Student's *t*‐test. (B) WB detected the protein levels of ACSL4, SLC7A11 and GPX4 in RKO cells at 6, 24 and 48 h after receiving radiotherapy (6 Gy). (C) Bar chart shows relative protein expression of ACSL4, SLC7A11 and GPX4 in RKO cells at 6, 24 and 48 h after receiving radiotherapy (6 Gy). *n* = 3, *p < 0.05 by ANOVA. (D) Lipid peroxidation level of RKO cells receiving radiotherapy (6 Gy). Bar charts show relative levels of lipid peroxidation in RKO cells. *n* = 3, ****p < 0.0001 by two‐tailed unpaired Student's *t*‐test. (E) Transmission electron microscopy images of RKO cells at 24 h received radiotherapy (6 Gy). Yellow arrows: mitochondria. (F) Lipid peroxidation levels were measured in RKO cells and RR cells at 24 h after radiotherapy (6 Gy). Bar chart shows relative levels of lipid peroxidation in RKO cells and RR cells. *n* = 3, **p < 0.01 by two‐tailed unpaired Student's *t*‐test. (G) qRT‐PCR analysis of PTGS2 in RKO cells and RR cells at 24 h after radiotherapy (6Gy). *n* = 3, *p < 0.05 by two‐tailed unpaired Student's *t*‐test. (H) WB detected the protein levels of ACSL4, SLC7A11 and GPX4 in RKO cells and RR cells at 24 h after receiving radiotherapy (6Gy). (I) Bar chart shows relative protein expression of ACSL4, SLC7A11 and GPX4 in RKO cells and RR cells at 24 h after receiving radiotherapy (6Gy). *n* = 3, **p < 0.01 by ANOVA.

The DEGs between RR and parental cells were significantly enriched in the ferroptosis pathway (Figure 1F). The heatmap shows the genes enriched in ferroptosis and cholesterol metabolism, including TF, MAP1LC3C, SLC40A1, SLC7A11, LIPG, APOA2, ANGPTL3, PCSK9, APOE and APOB (Figure 1G). To further confirm the association between ferroptosis and radiotherapy in CRC, the molecular and morphological features of ferroptosis were detected. Results showed that the expression of FRGs was significantly changed at the mRNA and protein levels after ionising radiation, including SLC7A11, GPX4, ACSL4 and PTGS2, in both RKO (Figure 2A–C) and DLD1 (Figure S2A–C) cells. Besides, irradiation resulted in an increase in lipid peroxidation in RKO (*p* < 0.0001, Figure 2D) and DLD1 (*p* < 0.0001, Figure S2D) cells. Additionally, transmission electron microscopy images showed that the mitochondria shrank significantly after radiation therapy, which is one of the features of ferroptosis [21] (Figure 2E). Altogether, radiotherapy can induce ferroptosis in CRC.

However, compared with parental cells, irradiation‐induced accumulation of lipid peroxidation in RR cells was significantly reduced (*p* = 0.0045, Figure 2F). Consistently, the expression of PTGS2 in RR cells was lower (*p* = 0.0011) than that in the parental cells after radiotherapy (Figure 2G). Moreover, some ferroptosis suppressor genes, such as SLC7A11 and GPX4, have also been found to be significantly overexpressed in RR cells (Figure 2H,I). Overall, these results illustrated that ferroptosis inactivation is one of the hallmarks of radioresistance in CRC.

### 3.3 Induction of Ferroptosis Restores the Radiation Sensitivity of CRC Cells

**FIGURE 3:**
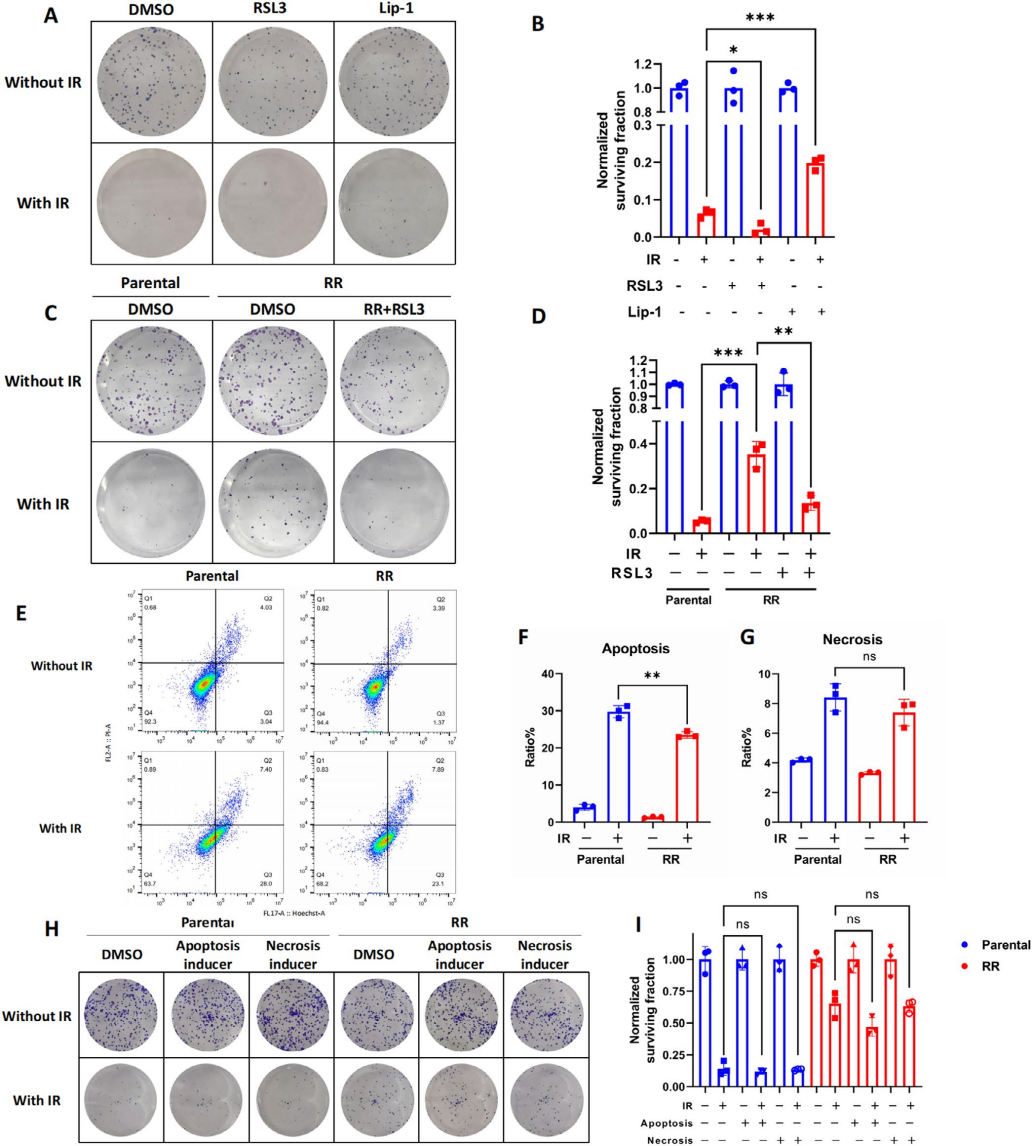
Induction of ferroptosis restores the radiation sensitivity of CRC cells. (A, B) Clonogenic survival of RKO cells pretreated with 100 nM RSL3, 5 μM lip‐1 or DMSO for 24 h followed by radiotherapy (4 Gy). The survival data were normalised to those of unirradiated cells. *n* = 3, ***p < 0.001,*p < 0.05 by two‐tailed unpaired Student's *t*‐test. (C, D) Clonogenic survival of RR cells that were pretreated with 100 nM RSL3 or DMSO for 24 h followed by radiotherapy (4 Gy). The survival data were normalised to those of unirradiated cells. *n* = 3, ***p < 0.001, **p < 0.01 by two‐tailed unpaired Student's *t*‐test. (E, F, G) Apoptosis and necrosis levels were measured in RKO and RR cells at 24 h after radiotherapy (6Gy). *n* = 3, **p < 0.01 by two‐tailed unpaired Student's *t*‐test. (H, I) Clonogenic survival of RKO and RR cells pretreated with apoptosis inducer (TNF‐α and SM‐164) and necrosis inducer (TNF‐α, SM‐164 and Z‐VAD‐FMK) for 24 h followed by radiotherapy (4 Gy). The survival data were normalised to those of unirradiated cells, *n* = 3.

It was found that ferroptosis was suppressed in CRC with a radioresistant phenotype, indicating the potential of ferroptosis inducers as an attractive strategy to combat radioresistance. At first, we treated RKO parental cells with the ferroptosis activator RSL3 and the suppressor Lip‐1. Results showed that RSL3 significantly enhanced the antitumor effects of radiation therapy in decreasing clonogenic survival (*p* = 0.0425), while Lip‐1 significantly attenuated this reduction (*p* = 0.0007) (Figure 3A,B). Then, RSL3 was used to treat RR cells, and it was observed that RSL3 sensitised RR cells to radiotherapy by the clonogenic cell survival assay (Figure 3C,D). In conclusion, these findings highlight that the induction of ferroptosis can restore the radiation sensitivity of CRC cells.

Radiotherapy not only induces ferroptosis but also apoptosis and necrosis in cells; we detected the changes in apoptosis and necrosis levels of RKO and RR cells after radiation by flow cytometry. It was found that compared with parental cells, less apoptosis was observed in RR cells after radiotherapy, while necrosis in RR cells remained no different (Figure 3E–G). What's more, RR cells were treated with apoptosis and necrosis inducers before receiving radiotherapy. However, they could not sensitise RKO and RR cells to radiotherapy by the clonogenic cell survival assay (Figure 3H,I).

What's more, we obtained gene expression and clinical data of CRC from the GEO database and investigated the predictive value of FRGs for response to chemoradiotherapy. The clinical characteristics of rectal cancer patients receiving chemoradiotherapy are detailed in Table 1. A 7‐FRGs signature (Table 2) was developed by lasso‐logistic regression analysis in the training cohort (Figure S3A,B). The predictive performance of this signature was evaluated by ROC curves, and the area under the ROC curve was 0.752 in the training cohort (95% CI: 0.653–0.852) (Figure S3C) and 0.861 in the testing cohort (95% CI: 0.751–0.971) (Figure S3D). Conclusively, FRGs can predict response to chemoradiotherapy in CRC patients. In addition, based on this 7‐FRGs signature, patients can be stratified into low‐ and high‐risk groups according to the optimal cut‐off value selected by the ROC curve. The low‐risk group had a longer DFS in both training (*p* = 0.012) (Figure S3E) and testing sets (*p* = 0.003) (Figure S3F). Longer cancer‐specific survival was observed in both training (*p* = 0.016) (Figure S3G) and testing sets (*p* = 0.02) (Figure S3H) as well. Taken together, FRGs can be used to predict radiosensitivity and prognosis in CRC.

**TABLE 1:** Clinical characteristics of rectal cancer patients receiving chemoradiotherapy.

	Response	Non‐response	p
Patients	91	51	
Age (median, range)	61.9 (36.2–81.4)	63.8 (35.7–81.5)	0.13
Gender (%)			
Male	66 (72.5%)	36 (70.6%)	0.85
Female	25 (27.5%)	15 (29.4%)	
Kras mutation (%)			
Yes	38 (41.8%)	21 (41.2%)	0.99
No	51 (56.0%)	29 (56.8%)	
Unknown	2 (2.2%)	1 (2.0%)	
Lymph node metastasis			
Yes	54 (59.3%)	32 (62.7%)	0.72
No	37 (40.7%)	19 (37.3%)	
Metastasis			
Yes	2 (2.2%)	3 (5.9%)	0.35
No	89 (97.8%)	48 (94.1%)	

**TABLE 2:** Seven genes in the prognostic model of rectal cancer patients receiving chemoradiotherapy.

Gene symbol	Gene name
JDP2	Jun dimerization protein 2
IFNG	Interferon gamma
ALOX12	Arachidonate 12‐lipoxygenase, 12S type
DNAJB6	DnaJ heat shock protein family (Hsp40) member B6
OTUB1	OTU deubiquitinase, ubiquitin aldehyde binding 1
MAPK14	Mitogen‐activated protein kinase 14
HERPUD1	Homocysteine inducible ER protein with ubiquitin like domain 1

### 3.4 Identification of NUPR1 Potential for Ferroptosis‐Mediated Radioresistance in CRC

**FIGURE 4:**
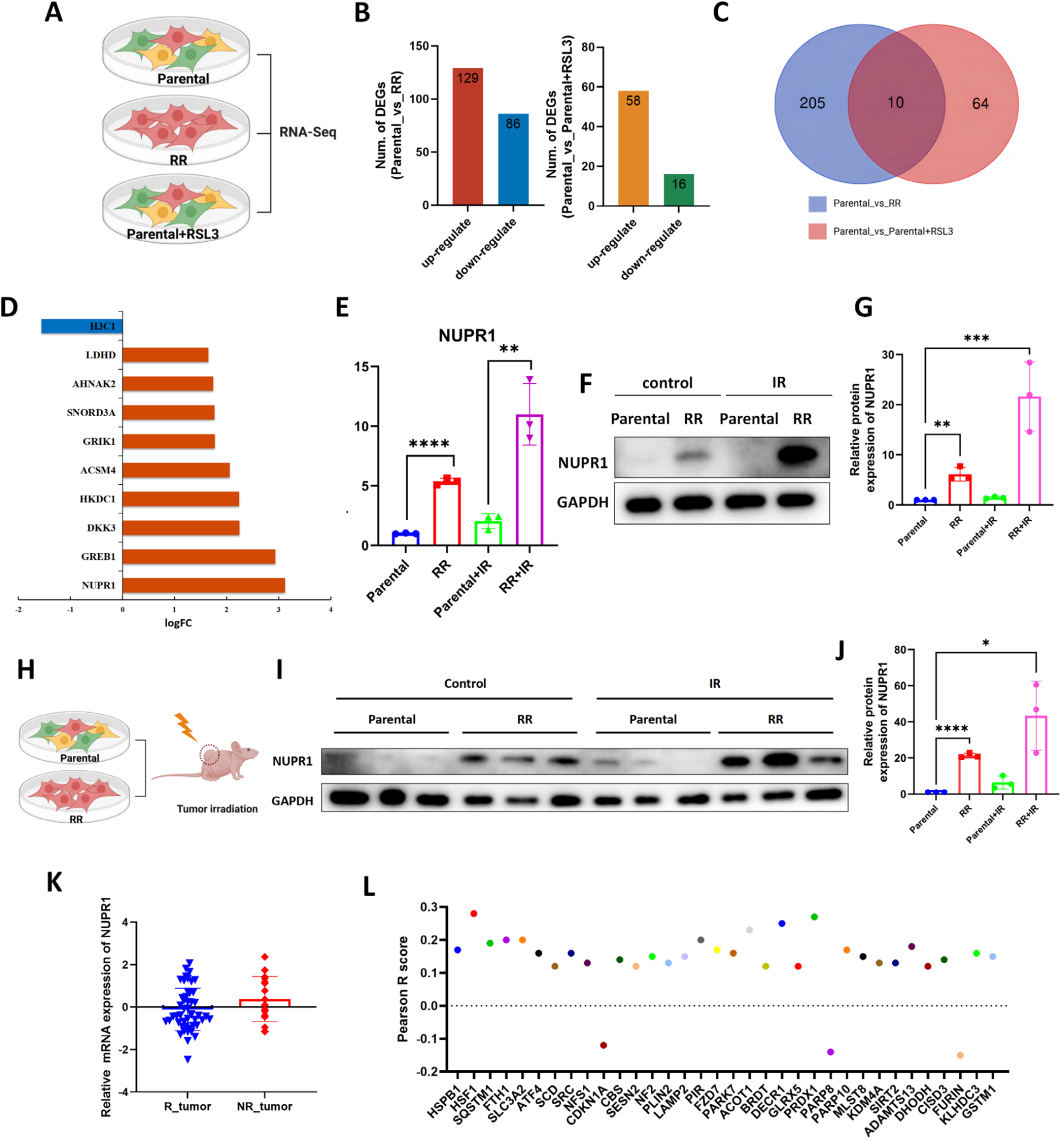
Identification of NUPR1 potential for ferroptosis‐mediated radioresistance in CRC. (A) Flow Chart of RNA Sequencing. (B) The DEGs between Parental cells and RR cells and DEGs between RKO cells and RKO cells treated with 1 μM RSL3. (C) Venn diagram to identify DEGs between parental cells and RR cells that were correlated with ferroptosis. (D) The differential expression of those 10 genes in RR cells and RKO cells. (E) qRT‐PCR analysis of NUPR1 in RKO cells and RR cells at 24 h after radiotherapy (6 Gy). *n* = 3, **p < 0.01, ****p < 0.0001 by two‐tailed unpaired Student's *t*‐test. (F) WB detected the protein levels of NUPR1 in RKO cells and RR cells at 24 h after radiotherapy (6 Gy). (G) Bar chart shows relative protein expression of NUPR1 in RKO cells and RR cells at 24 h after radiotherapy (6 Gy). *n* = 3, **p < 0.01, ***p < 0.001 by ANOVA. (H–I) WB detected the protein levels of NUPR1 in RKO cells and RR cells with or without radiotherapy in subcutaneous tumorigenesis model. (J) Bar chart shows relative protein expression of NUPR1 in RKO cells and RR cells with or without radiotherapy in subcutaneous tumorigenesis model. *n* = 3, *p < 0.05, ****p < 0.0001 by ANOVA. (K) The expression of NUPR1 in rectal cancer patients receiving neoadjuvant chemoradiation in the TCGA datasets. (L) The relationship between NUPR1 and ferroptosis suppressors.

In order to further understand molecular mechanisms of ferroptosis in regulating radioresistance, we performed RNA‐seq to compare gene expression between RKO parental and RR cells, and RKO cells pretreated with or without RSL3 (Figure 4A). In this way, the genes related to radioresistance and changed during ferroptosis were found. A total of 215 DEGs were identified between parental and RR cells, including 129 upregulated and 86 downregulated DEGs (Figure 4B). Besides, we obtained 74 DEGs between RKO cells with or without RSL3, of which 58 were significantly upregulated and 16 were significantly downregulated (Figure 4B). Then, we overlapped radioresistance‐related DEGs with ferroptosis‐related DEGs and found 10 DEGs common to two groups that might be engaged in ferroptosis‐mediated radioresistance (Figure 4C). Specifically, nine DEGs were upregulated, including LDHD, AHNAK2, SNORD3A, GRIK1, ACSM4, HKDC1, DKK3, GREB1 and NUPR1. In addition, H3C1 was downregulated (Figure 4D). Among them, NUPR1 was reported to be a transcriptional regulator with roles in cell cycle, DNA damage response, apoptosis, autophagy, and chromatin remodelling [41], and was of our great interest.

Firstly, the expression of NUPR1 in RR cells was investigated. It was shown that NUPR1 expression was higher in RR cells than in parental cells, and its expression was much higher in RR cells after radiotherapy (Figure 4E–G). What's more, we validated our results in the subcutaneous tumour model (Figure 4H) and found a consistent increase of NUPR1 in RR cells (Figure 4I,J). Additionally, we investigated its expression in rectal cancer patients receiving neoadjuvant chemoradiation in the TCGA data set and found a trend of higher expression of NUPR1 in nonresponse tumour (Figure 4K). Then, we explored its association with 69 ferroptosis suppressor genes [39] (Table S3) and found its significant association with 34 ferroptosis suppressor genes in CRC. Interestingly, 91.2% (31/34) of the genes were positively correlated with NUPR1 (Figure 4L). In sum, the expression of NUPR1 was elevated in radioresistant cells and might be involved in ferroptosis‐mediated radioresistance in CRC.

### 3.5 NUPR1 Promotes Radioresistance in CRC Cells by Inhibiting Ferroptosis

**FIGURE 5:**
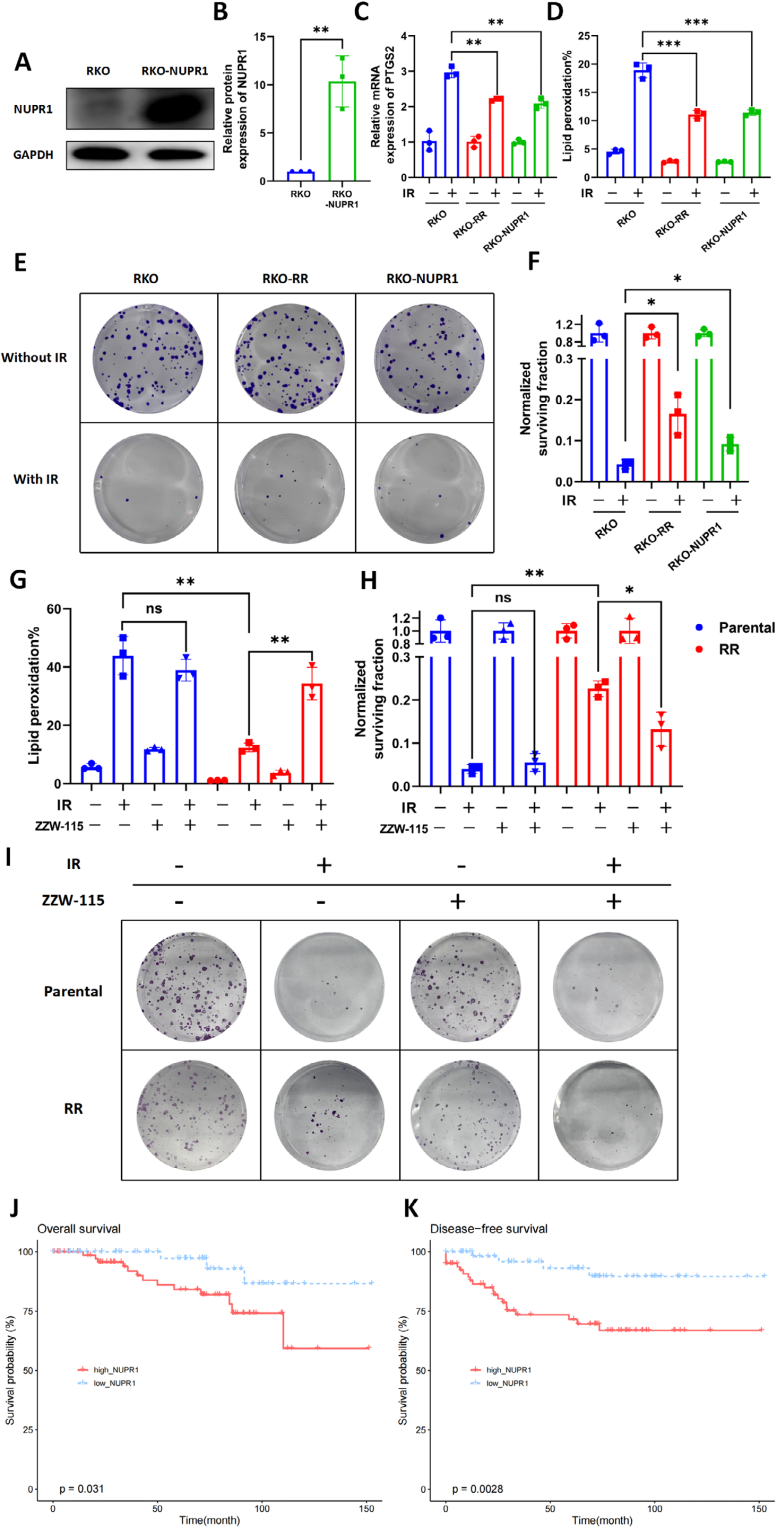
NUPR1 promotes radioresistance in CRC cells by inhibiting ferroptosis. (A) WB was used to verify the expression of NUPR1 in RKO cells and RKO‐NUPR1 cells. B Bar chart shows relative protein expression of NUPR1 in RKO cells and RKO‐NUPR1 cells. *n* = 3, **p < 0.01 by two‐tailed unpaired Student's *t*‐test. (C) Expression of PTGS2 in RKO cells, RR cells and RKO‐NUPR1 cells 24 h after radiotherapy (6 Gy). *n* = 3, **p < 0.01 by two‐tailed unpaired Student's *t*‐test. (D) Lipid peroxidation levels were measured in RKO cells, RR cells, and RKO‐NUPR1 cells at 24 h after radiotherapy (6 Gy). Bar chart shows relative levels of lipid peroxidation in RKO cells, RR cells and RKO‐NUPR1 cells. *n* = 3, ***p < 0.001 by two‐tailed unpaired Student's *t*‐test. (E, F) Clonogenic survival of RKO cells, RR cells and RKO‐NUPR1 cells receiving radiotherapy (4 Gy). The survival data were normalised to those of unirradiated cells. *n* = 3, *p < 0.05 by two‐tailed unpaired Student's *t*‐test. (G) Lipid peroxidation levels were measured in RKO cells and RR cells pretreated with 1 μM ZZW‐115 at 24 h after radiotherapy (6 Gy). Bar chart shows relative levels of lipid peroxidation in RKO cells, RR cells, and RKO‐NUPR1 cells. *n* = 3, **p < 0.01 by two‐tailed unpaired Student's *t*‐test. (H–I) Clonogenic survival of RKO cells RR cells and RR cells pretreated with 1 μM ZZW‐115 for 24 h followed by radiotherapy (4 Gy). The survival data were normalised to those of unirradiated cells. *n* = 3, **p < 0.01, *p < 0.05 by two‐tailed unpaired Student's *t*‐test. (J, K) Kaplan–Meier OS (I) and DFS (J) curves for patients in the high‐NUPR1 group (*N* = 81) and low‐NUPR1 group (*N* = 61).

To clarify the role of NUPR1 in ferroptosis‐regulated radioresistance in CRC, we constructed an NUPR1‐overexpressing CRC cell line (RKO‐NUPR1 and DLD1‐NUPR1) (Figure 5A,B, Figure S4A,B). The expression of PTGS2 in RKO‐NUPR1 cells was significantly lower (*p* = 0.0018) than that in control cells after radiotherapy (Figure 5C). Similarly, RKO‐NUPR1 cells and DLD1‐NUPR1 had lower lipid peroxidation levels (*p* = 0.0008, *p* = 0.0007) than control cells (Figure 5D, Figure S4C). Overall, NUPR1 can suppress ferroptosis as expected. More importantly, the clonogenic cell survival assay showed that RKO‐NUPR1 cells and DLD1‐NUPR1 cells formed many more colonies (*p* = 0.0136, *p* = 0.009) than the control cells after radiotherapy (Figure 5E,F and Figure S4D,E).

Besides, ZZW‐115, a strong NUPR1 inhibitor [42], was used to treat RR cells. Results showed that the level of lipid peroxidation in RR cells was significantly lower than in parental cells, but it can be increased (*p* = 0.0027) by ZZW‐115 (Figure 5G). Furthermore, the increased surviving colonies in RR cells after radiation exposure can be significantly attenuated by ZZW‐115 (*p* = 0.0203, Figure 5H,I), indicating that ZZW‐115 sensitises RR cells to radiotherapy.

**FIGURE 6:**
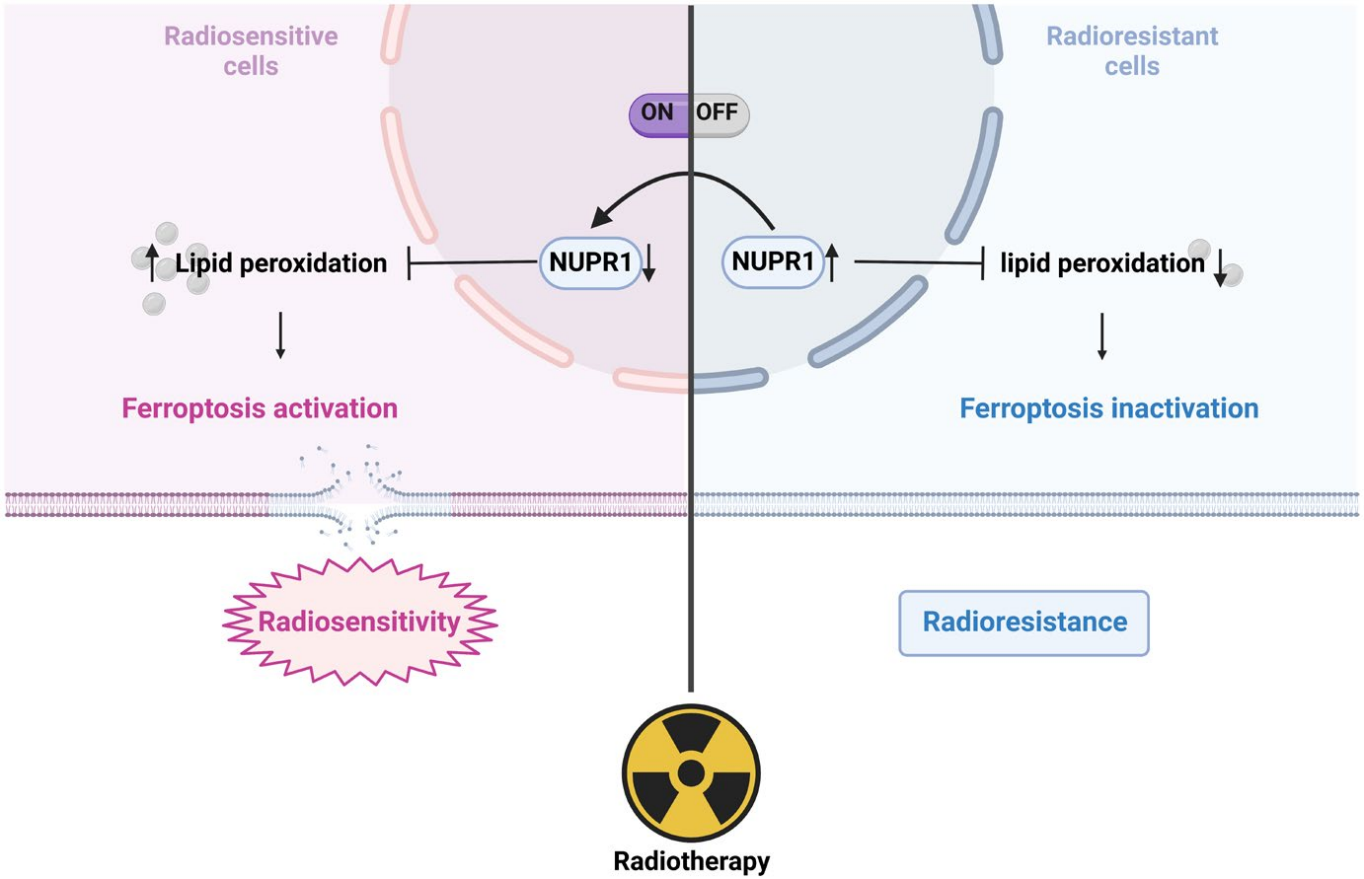
A working model of the mechanism by which NUPR1 can promote radioresistance in colorectal cancer cells by inhibiting ferroptosis.

In addition, we analysed the relationship between NUPR1 expression and patient prognosis from 142 rectal cancer patients who received neoadjuvant therapy in the GEO (GSE87211) database. We found that patients with high NUPR1 expression had significantly worse OS (*p* = 0.031, Figure 6J) and DFS (*p* = 0.0028, Figure 6K) compared to patients with low NUPR1 expression. Overall, the above results demonstrate that NUPR1 can promote radiation resistance of CRC cells by inhibiting ferroptosis, and targeting NUPR1 may be a potential strategy to relieve radioresistance associated with ferroptosis in CRC (Figure 6).

## 4 Discussion

Radiotherapy is one of the most common treatments for cancer. It mainly induces DNA damage in cells through high‐energy radiation. When these damages accumulate, it will lead to abnormal cell mitosis and cell death [9]. In the past, it was believed that apoptosis and necrosis were the main modes of radiation‐induced cell death [43, 44]. Some studies pointed out that tumour cells can resist radiotherapy by inhibiting radiation‐induced apoptosis through autophagy [45]. Recent studies have shown that ferroptosis also plays an important role in radiation‐induced cell death [17]. However, there is no clear conclusion on signatures of CRC with RR phenotype and the underlying mechanism of RR. In this study, we constructed a RR cell line by repeated radiotherapy. Although the RR cell line is polyclonal, it was confirmed that it is resistant to radiation. Upon conducting pathway enrichment analysis on the DEGs between RR and parental cells, we identified several enriched pathways, including transcriptional misregulation in cancer, metabolism, ferroptosis, and so on. Other researchers have found that in pancreatic cancer, radiotherapy can increase the glycolysis level of tumour cells, causing the accumulation of lactate in the tumour microenvironment, thereby increasing the activity of MDSCs and inhibiting sensitivity to radiotherapy [46]. Li et al. found that fatty acid oxidation in tumour cells promotes radiotherapy resistance in glioblastoma through immune evasion mediated by CD47 [47]. Ferroptosis has also been identified as one of the mechanisms through which radiotherapy eradicates tumour cells [18]. In this research, we mainly focused on the role of ferroptosis in tumour radioresistance. We found that ferroptosis inactivation is one of the hallmarks of RR in CRC. Besides, NUPR1 can promote the radiation resistance of CRC cells by inhibiting ferroptosis, and targeting NUPR1 may be a potential strategy to relieve radioresistance associated with ferroptosis in CRC. Other studies reported that ferroptosis suppressor genes are upregulated after radiotherapy, resulting in resistance to radiotherapy in lung cancer [17], which is consistent with our study.

Recently, the role of ferroptosis agonists in tumour treatments has been widely reported. For example, sorafenib can induce ferroptosis by inhibiting cystine‐glutamate transport receptors [25]; ferroptosis agonists, such as erastin and RSL3, can synergize with cisplatin or PD‐L1 monoclonal antibody to suppress tumour growth [29, 30]. Our study found that ferroptosis agonist RSL3 restored radiosensitivity of radioresistant CRC cells, making it a valuable target to improve the radiotherapy effect in CRC patients. Other researchers have also found that ferroptosis agonists have a radiosensitising effect. For example, Cobler et al. found that erastin can increase radiation sensitivity of breast cancer cells by inhibiting system Xc^−^ [48]. However, the clinical application of ferroptosis agonists is still very limited. Firstly, existing ferroptosis agonists have poor ability to target tumours and may also affect normal cells while killing tumours. Secondly, there are fewer drugs known to have the effect of promoting ferroptosis, which leads to fewer clinical choices. Thirdly, some ferroptosis agonists, such as erastin, have poor water solubility and metabolic instability, which limits their application in vivo [49]. Last, there is a notable absence of biomarkers that can accurately forecast a tumour's reaction to ferroptosis agonists, particularly those that can be identified directly from liquid biopsy specimens of patients [50]. Therefore, we still need to further explore the relationship between CRC radioresistance and ferroptosis to find the regulatory molecules that affect ferroptosis‐regulated radioresistance of CRC cells and then look for alternative treatment methods.

NUPR1 is a transcriptional regulator participating in cell cycle, DNA damage response, apoptosis, autophagy and chromatin remodelling [41]. NUPR1 was initially discovered in a rat model of pancreatitis [51], and subsequent studies have found that NUPR1 plays an important role in promoting tumorigenesis, tumour progression and even drug resistance [52, 53]. Under physiological conditions, the expression level of NUPR1 is relatively low in normal cells [54]. However, NUPR1 is activated under various stress conditions to protect cells [55]. Compared with normal cells, cancer cells have higher levels of basal cell stress. Therefore, the level of NUPR1 in cancer cells is correspondingly higher [56]. In the process of ferroptosis, a large amount of reactive oxygen species is released, leading cells to a stress state [20], which may also affect the expression of NUPR1. Recently, NUPR1 has been proved to be involved in regulating FRGs [43]. However, the exact role of NUPR1 in radiotherapy, radioresistance and ferroptosis is still unclear.

In this study, we found that NUPR1 promotes radioresistance of CRC cells by inhibiting ferroptosis. Recent studies have found that trifluoperazine, an antipsychotic drug, can inhibit NUPR1, and its effect has been confirmed in vitro experiments [57]. However, animal experiments found that trifluoperazine could affect the nervous system of mice, resulting in the inability of trifluoperazine to be used as an anticancer drug [42]. Subsequent studies found that ZZW‐115, a trifluoperazine derivative, can also inhibit NUPR1 without neurotoxicity [42]. Therefore, our study used ZZW‐115 to inhibit NUPR1 and found that it can restore the radiosensitivity of radioresistant CRC cells by promoting ferroptosis, which provides a new idea for reducing radioresistance in clinical practice. At present, although related studies have reported that NUPR1 can inhibit ferroptosis by transcriptionally regulating the expression of LCN2 in pancreatic cancer [43], promoting the initiation and development of tumours. However, whether NUPR1 plays a role in CRC radioresistance through the same mechanism requires further in‐depth study.

In conclusion, our study constructed a radioresistant CRC cell line and clarified the important role of ferroptosis in radioresistance in CRC. We found that NUPR1 is an important regulator of ferroptosis, which can promote the radioresistance of CRC cells by inhibiting ferroptosis. In our research, we utilised the databases from the GEO and MSK to corroborate our findings. Nonetheless, in public databases, obtaining certain data can be challenging because it may not be readily accessible or available for public use. Furthermore, the data in public databases often involves a wide range of populations and races, which leads to a very high level of data heterogeneity. Different populations and races may have significant differences in culture, lifestyle, genetic background and other aspects, which are reflected in the data, making the analysis and interpretation of the data more complex and challenging. Therefore, it would be better to supplement some clinical data from our centre to strengthen our conclusions. Moreover, the regulatory mechanism of NUPR1 expression and the specific molecular mechanisms by which NUPR1 inhibits ferroptosis and promotes radioresistance in CRC still need to be further explored. We will further investigate these issues in future research.

## Author Contributions

**Yimin Fang:** data curation (lead), investigation (lead), writing – original draft (lead). **Haiyan Chen:** conceptualization (equal), funding acquisition (equal), supervision (equal), writing – original draft (equal), writing – review and editing (lead). **Yunhua Liu:** supervision (equal), writing – review and editing (equal). **Kai Jiang:** methodology (equal), software (equal). **Yucheng Qian:** methodology (equal), software (equal). **Jingsun Wei:** investigation (equal), methodology (equal), software (equal). **Dongliang Fu:** formal analysis (equal), methodology (equal), resources (equal). **Hang Yang:** formal analysis (equal), investigation (equal). **Siqi Dai:** investigation (equal), methodology (equal), project administration (equal). **Tian Jin:** methodology (equal), software (equal). **Tongtong Bu:** funding acquisition (equal), project administration (equal), resources (equal). **Kefeng Ding:** supervision (equal), writing – review and editing (equal).

## Ethics Statement

All animal experiments were approved by the Institutional Ethics Committee of the Second Affiliated Hospital Zhejiang University School of Medicine (Zhejiang, P.R. China).

## Conflicts of Interest

The authors declare no conflicts of interest.

## Supporting information


**Figure S1.** Validation of radiation resistant cell lines and pathway enrichment analysis.
**Figure S2.** Radiotherapy induce ferroptosis in DLD1 cells.
**Figure S3.** FRGs can predict response to chemoradiotherapy in CRC patients.
**Figure S4.** NUPR1 promotes radioresistance in DLD1 cells.
**Table S1.** Primers for qRT‐PCR.
**Table S2.** 214 Ferroptosis‐related genes.
**Table S3.** 69 Ferroptosis suppressor genes.

## Data Availability

The data that support the findings of this study are openly available in figshare at https://doi.org/10.6084/m9.figshare.23994726, reference number 23994726.
