# Supplementary material for: NUPR1 Promotes Radioresistance in Colorectal Cancer Cells by Inhibiting Ferroptosis
Source: J Cell Mol Med. 2025 Apr 3;29(7):e70519. doi: 10.1111/jcmm.70519 (PMC11965884; doi:10.1111/jcmm.70519)
Supplement: Supplementary file 1 — Figure S1. Validation of radiation resistant cell lines and pathway enrichment analysis. Figure S2. Radiotherapy induce ferroptosis in DLD1 cells. Figure S3. FRGs can predict response to chemoradiotherapy in CRC patients. Figure S4. NUPR1 promotes radioresistance in DLD1 cells. Table S1. Primers for qRT‐PCR. Table S2. 214 Ferroptosis‐related genes. Table S3. 69 Ferroptosis suppressor genes. [file JCMM-29-e70519-s001.pdf]

Supplementary figure 1

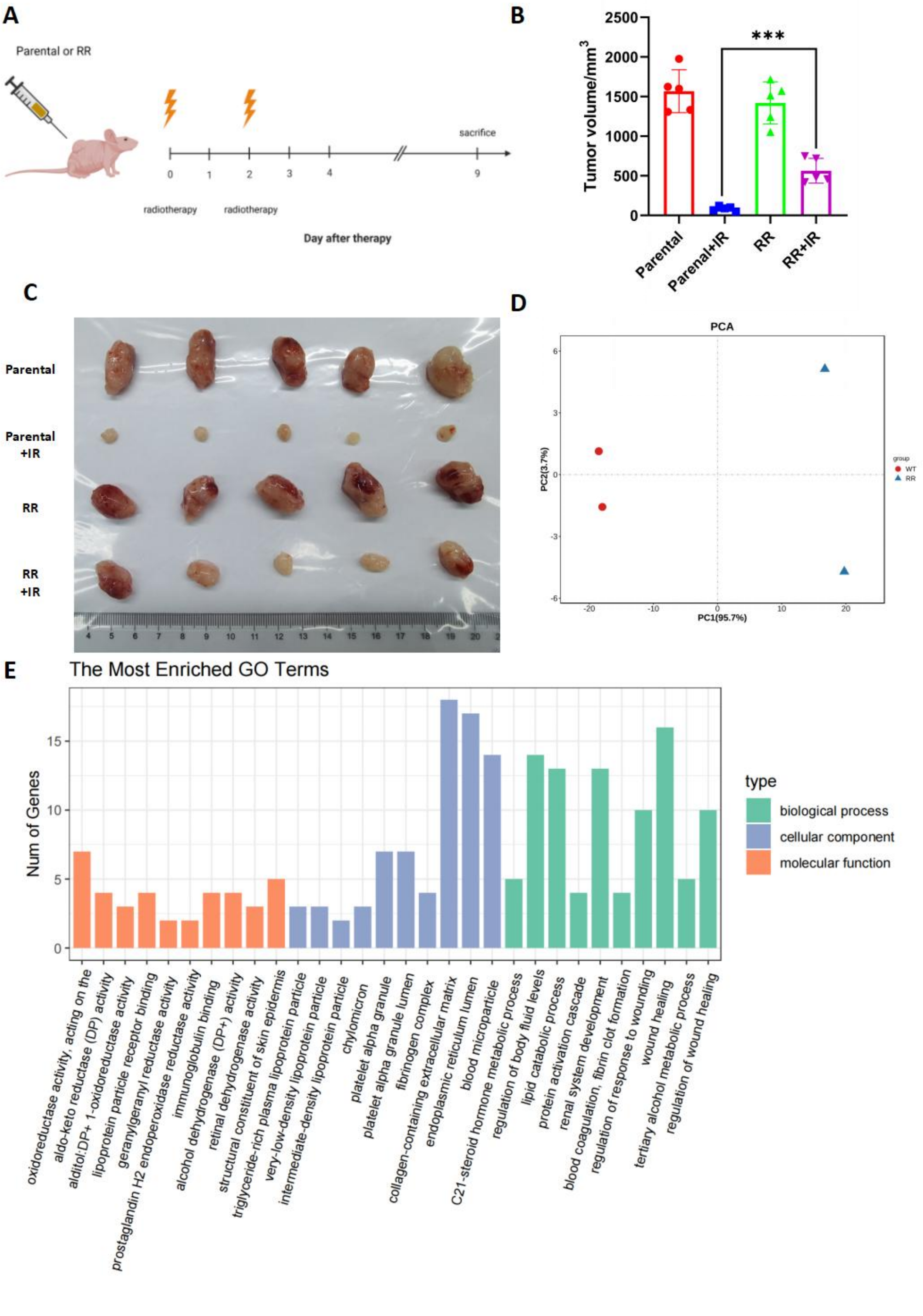

Fig. S1 Validation of radiation resistant cell lines and pathway enrichment analysis

A Experimental scheme of subcutaneous tumorigenesis with radiotherapy model. **B-C** In subcutaneous tumorigenesis model, RKO and RR tumor volume after radiotherapy (8Gy twice). The tumor volume was normalized to those of unirradiated tumors. n=5, $^*P < 0.05$,

** $P < 0.01$ by two-tailed unpaired Student's t-test. **D** PCA plot of RKO-parental cells and RKO-RR cells. **E** Gene Ontology (GO) enrichment analysis.

Supplementary figure 2

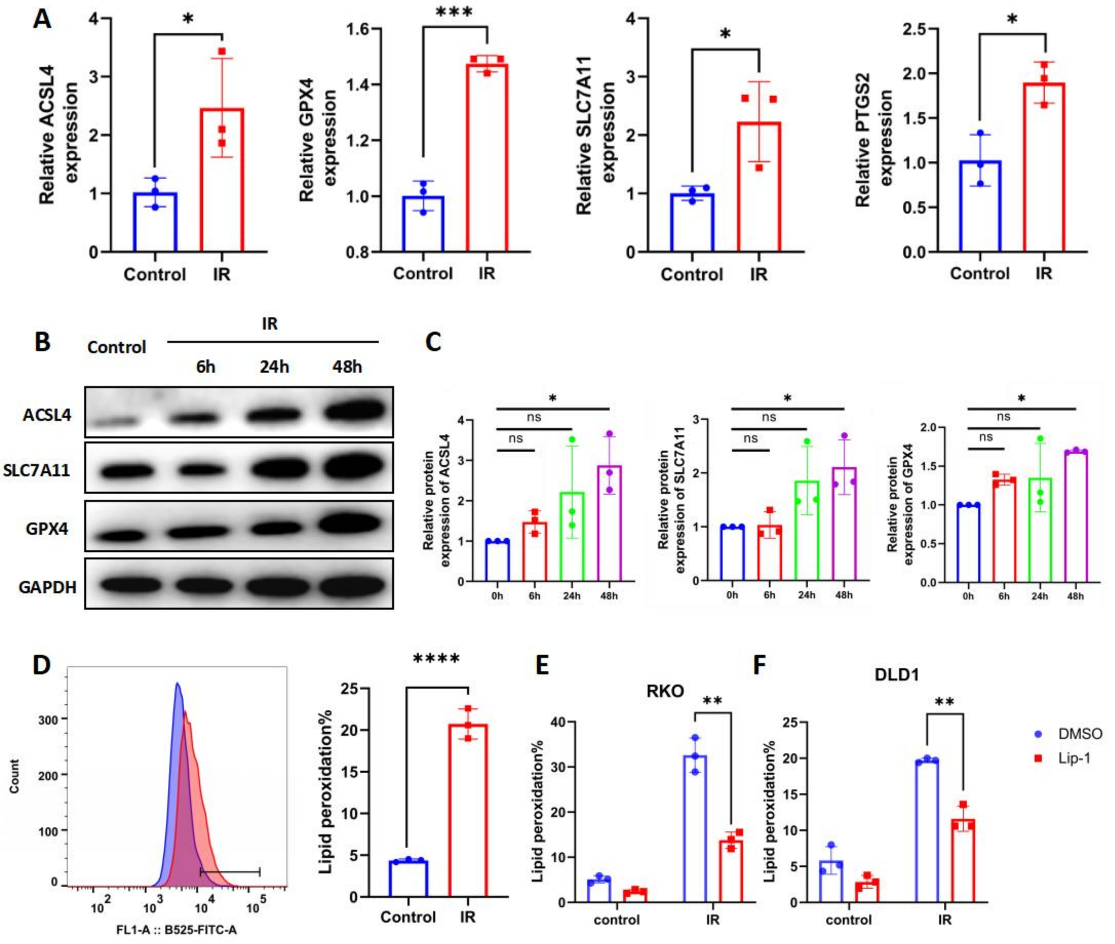

Fig. S2. Radiotherapy induce ferroptosis in DLD1 cells

A qRT-PCR analysis of ACSL4, GPX4, SLC7A11 and PTGS2 in DLD1 cells at 24 h after receiving radiotherapy (6Gy). n=3, $^{*}P < 0.05$, $^{***}P < 0.001$ by two-tailed unpaired Student's t-test. **B** WB detected the protein levels of ACSL4, SLC7A11, and GPX4 in DLD1 cells at 6 h, 24 h and 48 h after receiving radiotherapy (6Gy). **C** Bar chart shows relative protein expression of ACSL4, SLC7A11 and GPX4 in DLD1 cells at 6 h, 24 h and 48 h after receiving radiotherapy (6Gy). n=3, $^{*}P < 0.05$ by ANOVA. **D** Lipid peroxidation level of RKO cells receiving radiotherapy (6Gy). Bar charts show relative levels of lipid peroxidation in RKO cells. n=3, $^{****}P < 0.0001$ by two-tailed unpaired Student's t-test. **E-F** RKO cells and DLD1 cells were pretreated with 5 μM lip-1 or DMSO for 24 h and then received radiotherapy (6Gy), and lipid peroxidation levels were measured 24 h later. Bar charts show relative levels of lipid peroxidation in RKO cells and DLD1 cells. n=3, $^{**}P < 0.01$ by two-way

ANOVA.

Supplementary figure 3

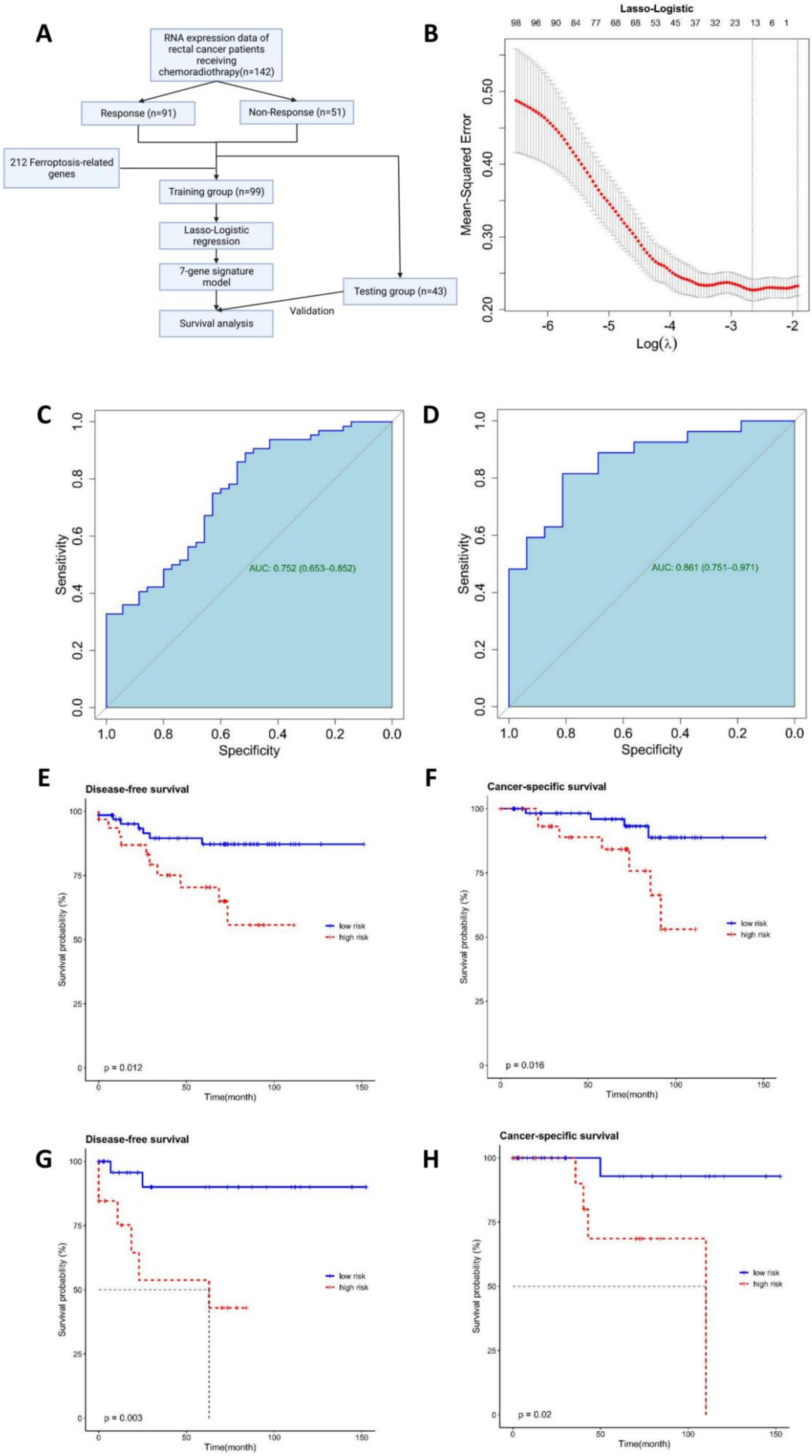

Fig. S3. FRGs can predict response to chemoradiotherapy in CRC patients

A Flow chart of data collection and analysis. **B** The generated coefficient distribution plots for the logarithmic (lambda) sequence for the selection of the best parameter (lambda). **C-D** The area under the ROC curve in training group was 0.752, and the area under the ROC curve in testing group was 0.861. **E-F** Kaplan – Meier DFS (E) and CSS (F) curves for patients in the high risk group and low risk group in training group. **G-H** Kaplan – Meier DFS (G) and CSS (H) curves for patients in the high risk group and low risk group in testing group.

Supplementary figure 4

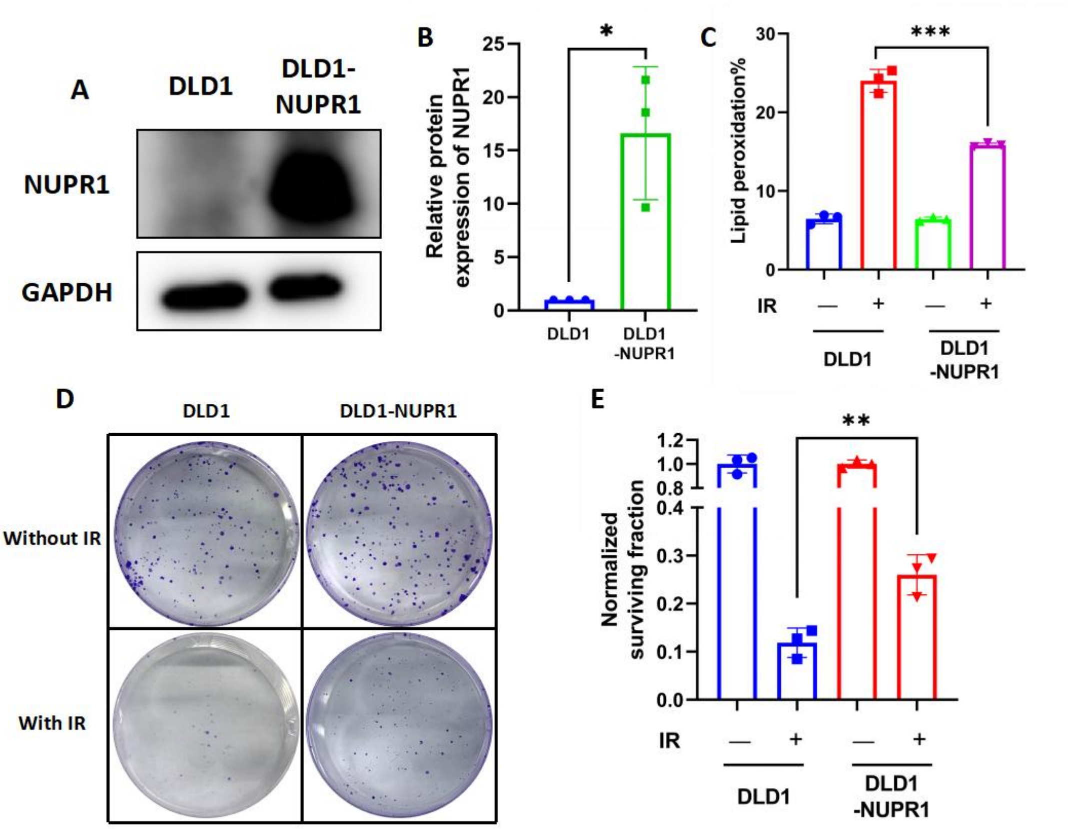

Fig. S4. NUPR1 promotes radioresistance in DLD1 cells

A WB was used to verify the expression of NUPR1 in DLD1 cells and DLD1-NUPR1 cells. **B** Bar chart shows relative protein expression of NUPR1 in DLD1 cells and DLD1-NUPR1 cells. n=3, $^{**}P < 0.01$ by two-tailed unpaired Student's t-test. **C** Lipid peroxidation levels were measured in DLD1 cells and DLD1-NUPR1 cells at 24 h after radiotherapy (6Gy). Bar chart shows relative levels of lipid peroxidation in DLD1 cells and DLD1-NUPR1 cells. n=3, $^{***}P < 0.001$ by two-tailed unpaired Student's t-test. **D-E** Clonogenic survival of DLD1 cells and DLD1-NUPR1 cells receiving radiotherapy (4Gy). The survival data were normalized to those of unirradiated cells. n=3, $^{**}P < 0.01$ by two-tailed unpaired Student's t-test.

Supplementary Table S1. Primers for qRT-PCR

gene		Sequence 5’→3’
SLC7A11	Forward	ATGCAGTGGCAGTGACCTTT
	Reverse	GGCAACAAAGATCGGAACTG
ACSL4	Forward	TGGAAGTCCATATCGCTCTGT
	Reverse	CCACAGCAAACCGTAGATGC
GPX4	Forward	GCCTTCCCGTGTAACCAGT
	Reverse	GCGAACTCTTTGATCTCTTCGT
DKK3	Forward	AGGTGTCATGGACTGTTGCC
	Reverse	ATCCAAGAACTACAGAAGAGCAG
PTGS2	Forward	GATCCCCAGGGCTCAAACAT
	Reverse	GAAAAGGCGCAGTTTACGCT
GREB1	Forward	ACATGACGAAGCAGAGGGTG
	Reverse	ACAGTGCTACTCACAAGATCC
NUPR1	Forward	CTCATCATGCCTATGCCCACT
	Reverse	TAGTGTCCATGGTCTGGCCT
β-actin	Forward	CGGAACCGCTCATTGCC
	Reverse	ACCCACACTGTGCCCATCTA

Supplementary Table S2. 214 Ferroptosis related genes

Number	Gene Symbol	Number	Gene Symbol	Number	Gene Symbol
1	ABCC1	40	CBS	79	GPT2
2	ACSF2	41	CD44	80	GPX2
3	ACSL3	42	CDKN1A	81	GPX4
4	ACSL4	43	CDKN2A	82	HBA1
5	ACVR1B	44	CDO1	83	HELLS
6	AGPAT3	45	CEBPG	84	HERPUD1
7	AIFM2	46	CHAC1	85	HIC1
8	AKR1C1	47	CHMP5	86	HIF1A
9	AKR1C2	48	CHMP6	87	HMGB1
10	AKR1C3	49	CISD1	88	HMOX1
11	ALB	50	CISD2	89	HNF4A
12	ALOX12	51	CS	90	HRAS
13	ALOX12B	52	CYBB	91	HSD17B11
14	ALOX15	53	DDIT3	92	HSF1
15	ALOX15B	54	DDIT4	93	HSPA5
16	ALOX5	55	DNAJB6	94	HSPB1
17	ALOXE3	56	DPP4	95	IDH1
18	ANGPTL7	57	DRD4	96	IFNG
19	ANO6	58	DRD5	97	IREB2
20	ARNTL	59	DUOX1	98	ISCU
21	ARRDC3	60	DUOX2	99	JDP2
22	ASNS	61	DUSP1	100	JUN
23	ATF3	62	EGFR	101	KEAP1
24	ATF4	63	EGLN2	102	KLHL24
25	ATG5	64	EIF2AK4	103	KRAS
26	ATG7	65	EIF2S1	104	LAMP2
27	ATM	66	ELAVL1	105	LINC00336
28	ATP5MC3	67	EMC2	106	LINC00472
29	ATP6V1G2	68	FADS2	107	LOC284561
30	AURKA	69	FBXW7	108	LOC390705
31	BAP1	70	FH	109	LONP1
32	BECN1	71	FTH1	110	LPCAT3
33	BLOC1S5-TXNDC5	72	FTL	111	LURAP1L
34	BNIP3	73	G6PD	112	MAFG
35	BRD4	74	GABPB1	113	MAP3K5
36	CA9	75	GCH1	114	MAPK1
37	CAPG	76	GDF15	115	MAPK14
38	CARS1	77	GLUT13	116	MAPK3
39	CAV1	78	GOT1	117	MIOX

118	MIR137	151	PHKG2	184	SNORA16A
119	MIR17	152	PLIN2	185	SOCS1
120	MIR30B	153	PLIN4	186	SQSTM1
121	MIR4715	154	PML	187	SRC
122	MIR6852	155	PRDX1	188	SRXN1
123	MIR9-1	156	PRDX6	189	STAT3
124	MIR9-2	157	PRKAA1	190	STMN1
125	MIR9-3	158	PRKAA2	191	TAZ
126	MT1G	159	PROM2	192	TF
127	MT3	160	PSAT1	193	TFRC
128	MTDH	161	PTGS2	194	TGFBR1
129	MUC1	162	RB1	195	TMBIM4
130	MYB	163	RGS4	196	TNFAIP3
131	NCF2	164	RIPK1	197	TP53
132	NCOA4	165	RPL8	198	TP63
133	NF2	166	RRM2	199	TRIB3
134	NFE2L2	167	SAT1	200	TSC22D3
135	NFS1	168	SCD	201	TUBE1
136	NGB	169	SELENOS	202	TXNIP
137	NNMT	170	SESN2	203	TXNRD1
138	NOS2	171	SETD1B	204	UBC
139	NOX1	172	SLC1A4	205	VDAC2
140	NOX3	173	SLC1A5	206	VEGFA
141	NOX4	174	SLC2A1	207	VLDLR
142	NOX5	175	SLC2A12	208	XBP1
143	NQO1	176	SLC2A14	209	YWHAE
144	NRAS	177	SLC2A3	210	YY1AP1
145	OTUB1	178	SLC2A6	211	ZEB1
146	OXSR1	179	SLC2A8	212	ZFP36
147	PANX1	180	SLC3A2	213	ZFP69B
148	PCK2	181	SLC40A1	214	ZNF419
149	PEBP1	182	SLC7A11		
150	PGD	183	SLC7A5		

Supplementary Table S3. 69 Ferroptosis suppressor genes

Number	Gene Symbol	Number	Gene Symbol
1	SLC7A11	36	ENPP2
2	GPX4	37	VDAC2
3	AKR1C1	38	FH
4	AKR1C2	39	CISD2
5	AKR1C3	40	MIR9-1
6	RB1	41	MIR9-2
7	HSPB1	42	MIR9-3
8	HSF1	43	CBS
9	GCLC	44	ISCU
10	NFE2L2	45	ACSL3
11	SQSTM1	46	OTUB1
12	NQO1	47	CD44
13	HMOX1	48	LINC00336
14	FTH1	49	BRD4
15	MUC1	50	PRDX6
16	SLC3A2	51	MIR17
17	MT1G	52	SESN2
18	SLC40A1	53	NF2
19	CISD1	54	ARNTL
20	FANCD2	55	HIF1A
21	FTMT	56	JUN
22	HSPA5	57	CA9
23	ATF4	58	TMBIM4
24	TP53	59	PLIN2
25	HELLS	60	MIR212
26	SCD	61	Fer1HCH
27	FADS2	62	AIFM2
28	SRC	63	LAMP2
29	STAT3	64	ZFP36
30	PML	65	PROM2
31	MTOR	66	CHMP5
32	NFS1	67	CHMP6
33	TP63	68	CAV1
34	CDKN1A	69	GCH1
35	MIR137		
